# Self-efficacy, organizational commitment, workload as predictors of life satisfaction in elementary school teachers: the mediating role of job satisfaction

**DOI:** 10.3389/fpsyg.2023.1066321

**Published:** 2023-05-31

**Authors:** Juan C. Cayupe, David Hugo Bernedo-Moreira, Wilter C. Morales-García, Fátima López Alcaraz, Karla Berenice Carrazco Peña, Jacksaint Saintila, Alcides Flores-Paredes

**Affiliations:** ^1^Unidad de Educación, Escuela de Posgrado, Universidad Peruana Unión, Lima, Peru; ^2^Escuela de posgrado, Programa de Doctorado en Educación, Universidad César Vallejo, Lima, Peru; ^3^Escuela de Medicina Humana, Facultad de Ciencias de la Salud, Universidad Peruana Unión, Lima, Peru; ^4^Facultad de Teología, Universidad Peruana Unión, Lima, Peru; ^5^Unidad de Salud, Escuela de Posgrado, Universidad Peruana Unión, Lima, Peru; ^6^Facultad de Medicina, Universidad de Colima, Colima, Mexico; ^7^Escuela de Medicina, Universidad Señor de Sipán, Chiclayo, Peru; ^8^Escuela Profesional de Educación Física, Universidad Nacional del Altiplano, Puno, Peru

**Keywords:** self-efficacy, teachers, life satisfaction, workload, organizational commitment, work engagement, job satisfaction

## Abstract

**Background:**

Life satisfaction as well as job satisfaction of teachers has a significant impact on educational outcomes.

**Objective:**

To evaluate a model of factors predicting life satisfaction through the mediating role of job satisfaction.

**Methods:**

This was a cross-sectional study, with a sample of 300 primary school teachers of both sexes (68% female, 32% male) and with a mean age of 42.52 years (SD = 10.04). They were administered the General Self-Efficacy Scale, the Satisfaction with Life Scale (SWLS), the Workload Scale (ECT), the Generic Job Satisfaction Scale, and the Organizational Commitment Questionnaire (OCQ). Structural equation modeling (SEM) was used for data analysis.

**Results:**

The SEM analysis found significant goodness-of-fit indices: (χ2 = 13.739; df = 5; *p* = <0.001; CFI = 0.99, TLI = 0.98, RMSEA = 0.05, SRMR = 0.04). Specifically, self-efficacy and organizational commitment were positive predictors of job satisfaction, while workload was a negative predictor of job satisfaction. The mediating effect of job satisfaction between self-efficacy, life satisfaction, workload, and overall life satisfaction was confirmed.

**Conclusion:**

The results confirm the importance of self-efficacy, organizational commitment, and workload in job satisfaction and overall life satisfaction of elementary education teachers. Job satisfaction acts as a mediator in this relationship. It is important to reduce workload and promote self-efficacy and organizational commitment to improve the well-being and satisfaction of teachers.

## 1. Introduction

In recent years, there has been increasing attention paid to the well-being of teachers, including their job and life satisfaction, as well as the stress and burnout they experience. Given that teachers face various challenges in their work, such as training, hiring, tenure, status, and working conditions (Schwarzer and Hallum, 2008; Lent et al., 2011), there has been a growing interest in the extent to which job satisfaction may be related to dispositional and job-related factors. Models have been developed that include different predictors to explain or predict teacher job satisfaction. These models may include self-efficacy, organizational commitment, and workload (Paloş et al., 2022). Based on Bandura’s (2010) social cognitive theory, Lent and Brown (2006) proposed a theory of job satisfaction that combines many components into a unified and empirically testable model. Social cognitive theory proposes a multifactorial model of job satisfaction that integrates different factors and explains how these factors contribute to job satisfaction and life satisfaction (Lent and Brown, 2006; Marcionetti and Castelli, 2022).

Self-efficacy is indirectly related to life satisfaction through job satisfaction. The feeling of having the ability to perform tasks and achieve work goals can help a person make progress in their teaching goals, which in turn is associated with life satisfaction (Lent and Brown, 2008). Similarly, job commitment can also have an indirect impact on life satisfaction through job satisfaction. Support and commitment to development as a teacher and the feeling of being valued by the school can help a person feel satisfied with their work, which in turn is associated with life satisfaction (Lent et al., 2011). On the other hand, teaching work in a competitive educational environment can generate stress and resistance to change if the challenges of change are not adequately explained. This resistance can have a negative effect on teachers’ motivation and job satisfaction (Michel et al., 2013; Avidov-Ungar and Magen-Nagar, 2014; Paloş et al., 2022). Therefore, workload is an important factor to consider in teachers’ life satisfaction. The literature suggests that workload can have a negative impact on job satisfaction and thus on teachers’ life satisfaction (Paloş et al., 2022). Additionally, job satisfaction has been a focus of attention for organizational and vocational researchers for a long time. Vocational psychology tends to be concerned with job satisfaction as an end in itself, while organizational psychology has been more concerned with the potential organizational consequences of job satisfaction, such as productivity, commitment, role withdrawal, and turnover (Fritzsche and Parrish, 2005).

The Lent and Brown model consists of five classes of predictor variables: personality/affective traits, participation in goal-directed activities, self-efficacy expectations, job conditions and supports, and environmental obstacles. The theory suggests that people are likely to be satisfied with their work when they feel they have the ability to perform tasks and achieve work goals (self-efficacy), are exposed to positive job conditions and rewards, perceive that they are making progress toward relevant goals, receive support for their goals, and possess personality traits that predispose them to experiencing positive mood (Lent et al., 2011).

In this sense, job satisfaction acts as a mediating factor between self-efficacy, organizational commitment, workload, and life satisfaction of basic education teachers.

### 1.1. Literature review

#### 1.1.1. Life satisfaction

Life satisfaction and job satisfaction are important factors to consider in the study of teachers’ well-being. Life satisfaction is associated with personal, psychological, and social positive outcomes, while job satisfaction is influenced by work environment characteristics and personality (Marcionetti and Castelli, 2022). Life satisfaction is based on the subjective evaluation of one’s own life and is influenced by health, work, economic income, spirituality, and leisure activities (Gilman and Huebner, 2000; Diener and Tay, 2012). In addition, it has been shown that teachers who are satisfied with their life in general also tend to be more satisfied with their job (Lent et al., 2001). Teachers’ job satisfaction has been related to teacher retention and relationships with students (Veldman et al., 2013). Teachers with higher job satisfaction are better educational agents, and their work mentality, attitudes, values, and ideas are related to their work and to an individual and collective vision (Ignat and Clipa, 2012). Therefore, it is important to highlight that although job satisfaction can be a positive indicator of teachers’ well-being, burnout is undoubtedly a negative indicator. Therefore, multifactorial studies on teachers’ well-being are necessary, considering different personal and contextual variables as predictors of both burnout and job and life satisfaction. The integrative model (Lent and Brown, 2006) offers a useful tool for examining the relationships between different factors that influence teachers’ job satisfaction and life satisfaction.

#### 1.1.2. Self-efficacy

Self-efficacy, or the belief in one’s own ability to perform a task, is an important factor in teachers’ job satisfaction. It has been shown that self-efficacy is positively related to motivation and job performance in teaching (Skaalvik and Skaalvik, 2007). Moreover, self-efficacy is related to teachers’ job satisfaction (Brown et al., 1996; Connolly and Viswesvaran, 2000; Judge et al., 2002). Self-efficacy has also been related to progress towards personal work goals, which in turn is related to job satisfaction and life satisfaction (Lent et al., 2011). Self-efficacy is perceived as an important component of individuals’ work well-being and determines the motivation and effort devoted to tasks (Moriano et al., 2012). Studies have shown that self-efficacy is related to teachers’ job satisfaction, especially when measured in terms of specific tasks or goals (Judge and Larsen, 2001; Caprara and Steca, 2006; Badri et al., 2013). The relationship between progress towards work goals and job satisfaction has also been demonstrated (Judge et al., 2005; Wiese and Freund, 2005). Furthermore, organizational support, promotion, positive student behavior, psychological capital, and work conditions also have significant effects on job satisfaction and well-being in the teaching profession (Yoo and Rho, 2020; Ortan et al., 2021). Therefore, self-efficacy, along with other factors, influences teachers’ organizational commitment, which in turn is positively related to job satisfaction (Skaalvik and Skaalvik, 2009). Thus, self-efficacy is an important factor in teachers’ job satisfaction and well-being, and its relationship with other variables has been studied (Katsantonis, 2019; Hajovsky et al., 2020; Cataudella et al., 2021; Salas-Rodríguez et al., 2021; Wettstein et al., 2021).

#### 1.1.3. Organizational commitment

Organizational commitment is defined as the individual’s adherence to the goals and values of the organization, as well as their willingness to make an extra effort to achieve those goals (Badri et al., 2013). Teachers’ organizational commitment has been positively related to job satisfaction (Skaalvik and Skaalvik, 2009). Furthermore, organizational commitment is a psychological attitude of members of an organization and their attachment to the workplace, which influences retention and passion to achieve goals (Kotzé and Nel, 2020). Three types of organizational commitment have been identified in teachers: to the school (UNESCO, 2021), to the profession (Lin et al., 2019), and to the students (Jara and Dagach, 2014). Meyer and Allen propose a conceptualization of organizational commitment divided into three components: affective (emotional attachment), continuance (desire to stay in the organization), and normative (sense of duty to stay in the organization) (Sezen-Gultekin et al., 2021). These factors influence life satisfaction and job performance (Wayoi et al., 2021), job satisfaction, academic performance, and self-efficacy (Park, 2007; Dee et al., 2016).

#### 1.1.4. Workload

Perceived workload is related to emotional exhaustion and burnout (Alarcon, 2011). Some studies indicate that perceived workload is not related to job satisfaction after controlling for the effects of role ambiguity and role overload (Bowling et al., 2015). However, perceived workload is relevant to teacher well-being and is expected to predict self-efficacy, job satisfaction, and burnout (Goh et al., 2015).

Some studies have found that high levels of workload are negatively related to job satisfaction (Skaalvik and Skaalvik, 2007), while others have found mixed results regarding the relationship between workload and teacher job satisfaction (Hakanen et al., 2006). If workload is too high, it can generate stress and reduce job satisfaction (Bakker and Bal, 2010), while an appropriate workload can contribute to job satisfaction and job performance (Bakker and Demerouti, 2017). The literature has explored the workload of teachers and has shown that being a teacher is hard work and requires coping with a considerable amount of adverse events (Bauer et al., 2007). Moreover, when work demands do not match the capabilities or needs of the individuals who are going to perform the tasks, that is when health impairments occur (Voydanoff, 2004). The scope (Xiao et al., 2011). Negative or threatening events related to the school increase workload. Too high or low mental workload will result in decreased teacher work capacity, while moderate mental workload will maintain and stabilize their work capacity (Xiao et al., 2011, 2015). In addition, reducing workload and the resulting decrease in the student-teacher ratio could reduce teacher stress (Hojo, 2021), while workload indirectly influences life satisfaction through job satisfaction (Gardner and Parkinson, 2011).

#### 1.1.5. Job satisfaction

Lent, Brown, and Hackett’s social cognitive career theory (Lent et al., 1994) consider self-efficacy as a central component of job satisfaction and point out that progression toward work goals and perception of organizational support are also important factors. Thus, teacher job satisfaction is influenced by their life satisfaction and organizational commitment, which refers to their adherence to the organization’s goals and values and their willingness to make an extra effort to achieve them (Mowday and Steers, 1979).

Job satisfaction is defined as an employee’s affective reactions to a job based on a variety of elements (Pecino-Medina et al., 2015; Jiménez Figueroa et al., 2020). Job satisfaction is composed of intrinsic and extrinsic characteristics. Intrinsic factors include achievement, recognition, responsibility, advancement, growth, and the job itself, while extrinsic factors include supervision, working conditions, co-workers, pay policies, procedures, status, and personal life (Rahman et al., 2019). The literature shows that the link between job satisfaction and life satisfaction could be bidirectional, as life satisfaction can predict positive affect, goal attainment, efficacy support, and work conditions (Marcionetti and Castelli, 2022). Therefore, job satisfaction is reciprocally related to life satisfaction and posits several paths between the precursors of job satisfaction (Lent and Brown, 2006).

The scarcity of studies in Peru on the job satisfaction of basic education teachers is evident. Therefore, it is important to identify the effects of self-efficacy, organizational commitment, and workload on life satisfaction, as well as the mediating role of job satisfaction in these effects. It is postulated that teachers who are able to achieve their work goals (self-efficacy), are committed to their work due to the work environment and conditions (organizational commitment), and have a sustainable workload will have higher life satisfaction.

**Figure 1:**
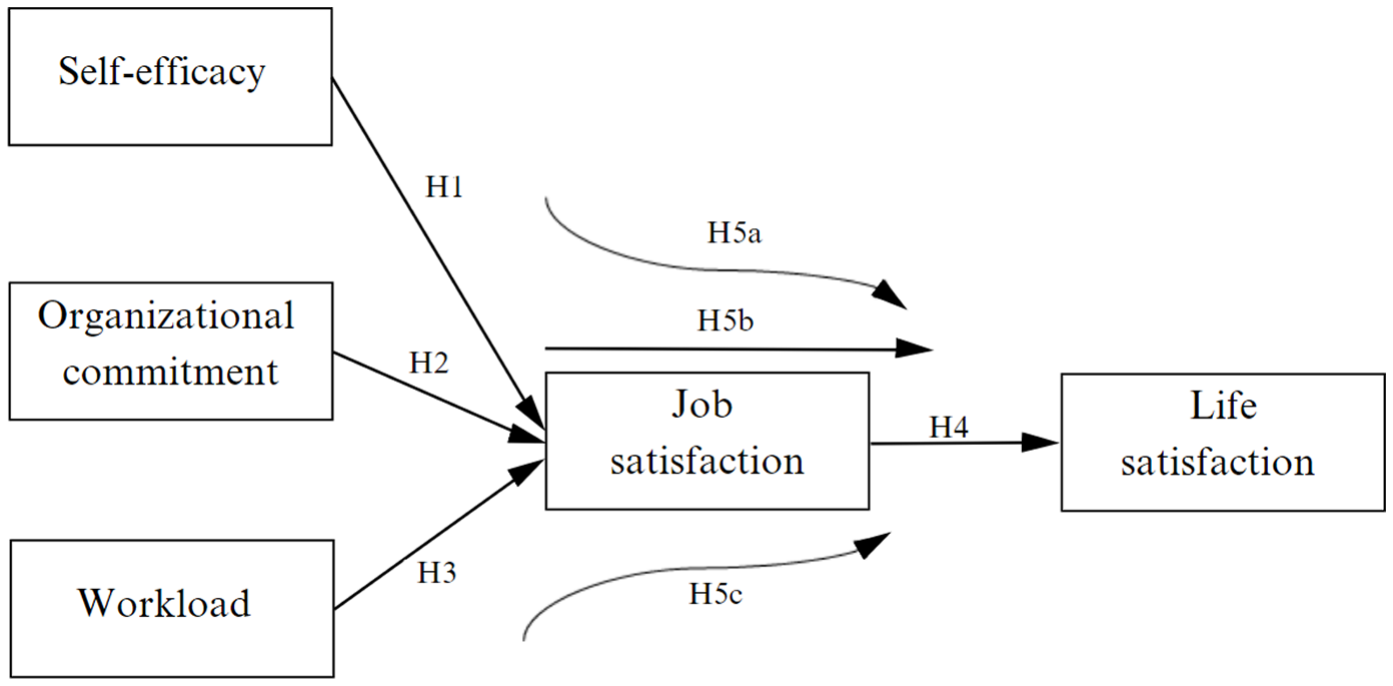
Proposed model.

Based on the literature review, the following hypotheses are proposed (Figure 1):

*H1*: There is a positive relationship between self-efficacy and job satisfaction.

*H2*: There is a positive relationship between organizational commitment and job satisfaction.

*H3*: There is a negative relationship between workload and job satisfaction.

*H4*: There is a positive relationship between job satisfaction and life satisfaction.

*H5a*: Job satisfaction mediates the relationship between self-efficacy and job satisfaction.

*H5b*: Job satisfaction mediates the relationship between organizational commitment and job satisfaction.

*H5c*: Job satisfaction mediates the relationship between workload and job satisfaction.

## 2. Materials and methods

### 2.1. Study design and population

A cross-sectional and explanatory study was designed considering latent variables represented by a system of structural equations (Ato et al., 2013). For the sample size, the effect size was analyzed by means of the Soper electronic calculator that considers the number of observed and latent variables in the model, the anticipated effect size (λ = 0.3), the desired statistical significance (α = 0.05), and the statistical power level (1−β = 0.95) (Soper, 2021). Inclusion criteria were: a) with a bachelor’s or master’s degree, b) basic education teachers (pre-school, primary and secondary), c) from public or private institutions, and d) with more than 3 months working in the institution. A total of 300 teachers from 4 educational institutions conveniently selected from the three regions (coast, highlands, and jungle) of Peru participated. Requests were sent to the principals of the educational institutions, indicating the purpose of the research. Once the permits were obtained from the 4 educational institutions, they were sent through various platforms to the teachers. Prior to data collection, the guidelines stipulated in the Declaration of Helsinki and the rules of confidentiality were considered by informing participants about the nature of the project, followed by obtaining informed consent. Data collection was carried out during the months of November 2021 and May 2022.

### 2.2. Ethical considerations

Prior to data collection, compliance with ethical standards was ensured. The participants were informed about the objective of the study, confidentiality, and ethics, and were told that their participation was voluntary; in addition, informed consent was obtained from the participants. The study was evaluated by the Research Ethics Committee of Universidad Peruana Unión (Cod. 2021-CE-EPG-000067). The research was carried out considering the guidelines stipulated in the Declaration of Helsinki.

### 2.3. Instruments

#### 2.3.1. Satisfaction with life

It was evaluated through the Satisfaction with Life Scale (SWLS) in its version for the Peruvian population (Calderón-de et al., 2018). This instrument evaluates the degree of overall life satisfaction and is composed of 5 items, with 5 Likert-type items: Strongly Disagree = 1; Slightly Disagree = 2; Neither Agree nor Disagree = 3; Slightly Agree = 4; Strongly Agree = 5. Furthermore, in this study, the model presented adequate reliability indices on the total scale (*α* = 0.88, *ω* = 0.86, *H* = 0.88), and the model presented adequate validity indices (*χ*^2^ = 18.154; df = 5; *p* = <0.001; CFI = 0.99, TLI = 0.99, RMSEA = 0.06, SRMR = 0.02).

#### 2.3.2. Job satisfaction

It was evaluated with the Generic Job Satisfaction Scale (Salessi and Omar, 2018), The survey is composed of 7 items, with a 5-point Likert-type response, ranging from 1 = Never; 2 = Almost never; 3 = Sometimes; 4 = Almost always; 5 = Always, where the constructs of trust, commitment and organizational cynicism are measured. Furthermore, in this study, the model presented adequate reliability indices (*α* = 0.88, *ω* = 0.89, *H* = 0.93), and the model presented adequate validity indices (*χ*^2^ = 41.411; df = 9; p = <0.001; CFI = 0.99, TLI = 0.99, RMSEA = 0.07, SRMR = 0.03).

#### 2.3.3. Organizational commitment

The Organizational Commitment Questionnaire (OCQ) was used (Díaz et al., 2006), which is used to measure the level of work engagement, the desire to remain in the organization, maintain high levels of effort, and acceptance of organizational goals and values. This scale is made up of 9 items and is composed of 5 Likert-type items: Strongly disagree = 1; Disagree = 2; Neither agree nor disagree = 3; Agree = 4; Strongly agree = 5. Furthermore, in this study, the model presented adequate reliability indices for dimension 1 (*α* = 0.98, *ω* = 0.88, *H* = 0.92) and dimension 2 (*α* = 0.98, *ω* = 0.88, *H* = 0.91), and the model presented adequate validity indices (*χ^2^* = 68.586; df = 13; *p* = <0.001; CFI = 0.99, TLI = 0.99, RMSEA = 0.07, SRMR = 0.03).

#### 2.3.4. Self-efficacy

The General Self-Efficacy Scale was used (Cid et al., 2010). It is a self-report instrument where the person responds to each item according to what he/she perceives of his/her capacity at the moment. It consists of 10 items, with a minimum score of 10 points and a maximum of 40. The responses are Likert-type: Incorrect (1 point); Barely true (2 points); Rather true (3 points); True (4 points). In this scale, the higher the score, the higher the perceived general self-efficacy. In this study, the model presented adequate reliability indices in the total scale (α = 0.88, ω = 0.92, H = 0.96), and the model presented adequate validity indices (*χ*^2^ = 174.899; df = 35; *p* = <0.001; CFI = 0.99, TLI = 0.99, RMSEA = 0.07, SRMR = 0.04).

#### 2.3.5. Workload

The Workload Scale (ECT) (Calderón et al., 2018), which examines quantitative and qualitative workload and is composed of 6 items and items that are randomly distributed with respect to their content, and presented in ordinal format with five response options ranging from 0 (Never); 1 (Almost never); 2 (Sometimes); 3 (Quite often); 4 (Very often: every day). A single score is obtained from the simple sum of the responses to the items. Furthermore, in this study, the model presented adequate reliability indices on the total scale (*α* = 0.88, *ω* = 0.75, *H* = 0.80) and the model presented adequate validity indices (*χ*^2^ = 13.739; df = 5; *p* = <0.001; CFI = 0.99, TLI = 0.98, RMSEA = 0.05, SRMR = 0.04).

### 2.4. Statistical analysis

Descriptive statistics were calculated, such as mean (M), standard deviation (SD), skewness (g1), and kurtosis (g2) considered with values ±2 (Pérez and Medrano, 2010; Gravetter and Wallnau, 2014). The normality of data distribution was verified using the multivariate estimation of Mardia, where values less than 5 indicate normality, and subsequently, a bivariate analysis was conducted to check the relationships between variables.

Subsequently, structural equation modeling (SEM) was applied, which allowed evaluating the direct and mediator effects of latent predictor variables on outcome variables (Byrne, 2001). The R 4.1.2 program and the “lavaan” library were used, which offers a WLSMV estimator for non-normal data (Kline, 2016). Since the Chi-square test is sensitive to sample size (West et al., 2012), goodness of fit indices, comparative fit indices (CFI) and Tucker-Lewis indices (TLI) that range between 0.90 and 0.95 would indicate an acceptable fit, and values above 0.95 would indicate a good fit. Root mean square error of approximation (RMSEA) and standardized root mean square residual (SRMR) indices with values between 0.05 and 0.08 would indicate an acceptable fit, and values below 0.05 would indicate a good fit following the proposals of Escobedo et al. and Rex (Escobedo et al., 2016; Kline, 2016).

Regarding the evaluation of mediation, the “psych” and “mediation” packages were used (Kotzé and Nel, 2020). Mediation was evaluated considering that it indicates that the mediator variable (M) must be causally located between the independent variable (X) and dependent variable (Y), and that the indirect effect of X on Y occurs through M Y (Baron and Kenny, 1986; Hayes and Rockwood, 2017). Therefore, bootstrapping with 5,000 interactions was applied to test the indirect effect.

## 3. Results

### 3.1. Sociodemographic data

A total of 300 Peruvian teachers participated (Table 1), with a mean age of 42.52 years (SD = 10.04). The majority were female (68.0%), hired (46.0%), from private institutions (70.3%), from the coastal region (76.0%), and working at the secondary level (39.7%).

**Table 1:** Distribution of sociodemographic variables.

Characteristics	*n* (%)
*Age groups (years)*	
21–36	81 (27)
37–51	159 (53)
52–67	60 (20)
*Sex*	
Female	204(68.0)
Male	96(32.0)
*Occupational group*	
Contracted	138(46.0)
Appointed	49(16.3)
Employee	113(37.7)
*Type of institution*	
Private	211(70.3)
Public	89(29.7)
*Region*	
Coast	228(76.0)
Highlands	48(16.0)
Jungle	24(8.0)
*Occupational level*	
Initial	50(16.7)
Primary	131(43.7)
Secondary	119(39.7)

### 3.2. Preliminary analysis

Table 2 shows the descriptive statistics and correlations of the study variables. The analyses between the variables studied yielded highly significant correlation coefficients (*p* < 0.01). The bivariate analysis indicated a positive relationship between life satisfaction and job satisfaction (r = 0.41, *p* < 0.01), self-efficacy (r = 0.33, *p* < 0.01), and organizational commitment (r = 0.43, *p* < 0.01), while a negative relationship was found with workload (r = −0.22, *p* < 0.01). Furthermore, job satisfaction was positively correlated with self-efficacy (r = 0.42, *p* < 0.01) and organizational commitment (r = 0.68, *p* < 0.01), and negatively correlated with workload (r = −0.34, *p* < 0.01). Self-efficacy also showed a positive correlation with organizational commitment (r = 0.34, *p* < 0.01) and a negative correlation with workload (r = −0.53, *p* < 0.01). Finally, organizational commitment was negatively correlated with workload (r = −0.22, *p* < 0.01). However, the data revealed multivariate kurtosis, as the Mardia normalized estimate was 35.35, so the WLSMV estimator, which is robust for analyzing data that are not normally distributed, was applied.

**Table 2:** Descriptive statistics and correlation matrix of the study variables.

Variable	1	2	3	4	Mean	SD	g^1^	g^2^
1. Satisfaction with life	–				20.14	3.43	−0.83	0.98
2. Job satisfaction	0.41**	–			25.18	3.49	−0.64	0.12
3. Self-efficacy	0.33**	0.42**	–		33.60	5.21	−0.57	−0.56
4. Organizational commitment	0.43**	0.68**	0.34**	–	28.51	4.43	−0.54	0.23
5. Workload	−0.22**	−0.34**	−0.29**	−0.22**	8.42	2.60	0.28	0.42

SD, standard deviation; g^1^ = skewness; g^2^ = kurtosis. **p < 0.01.

### 3.3. Analysis of the structural model

**Figure 2:**
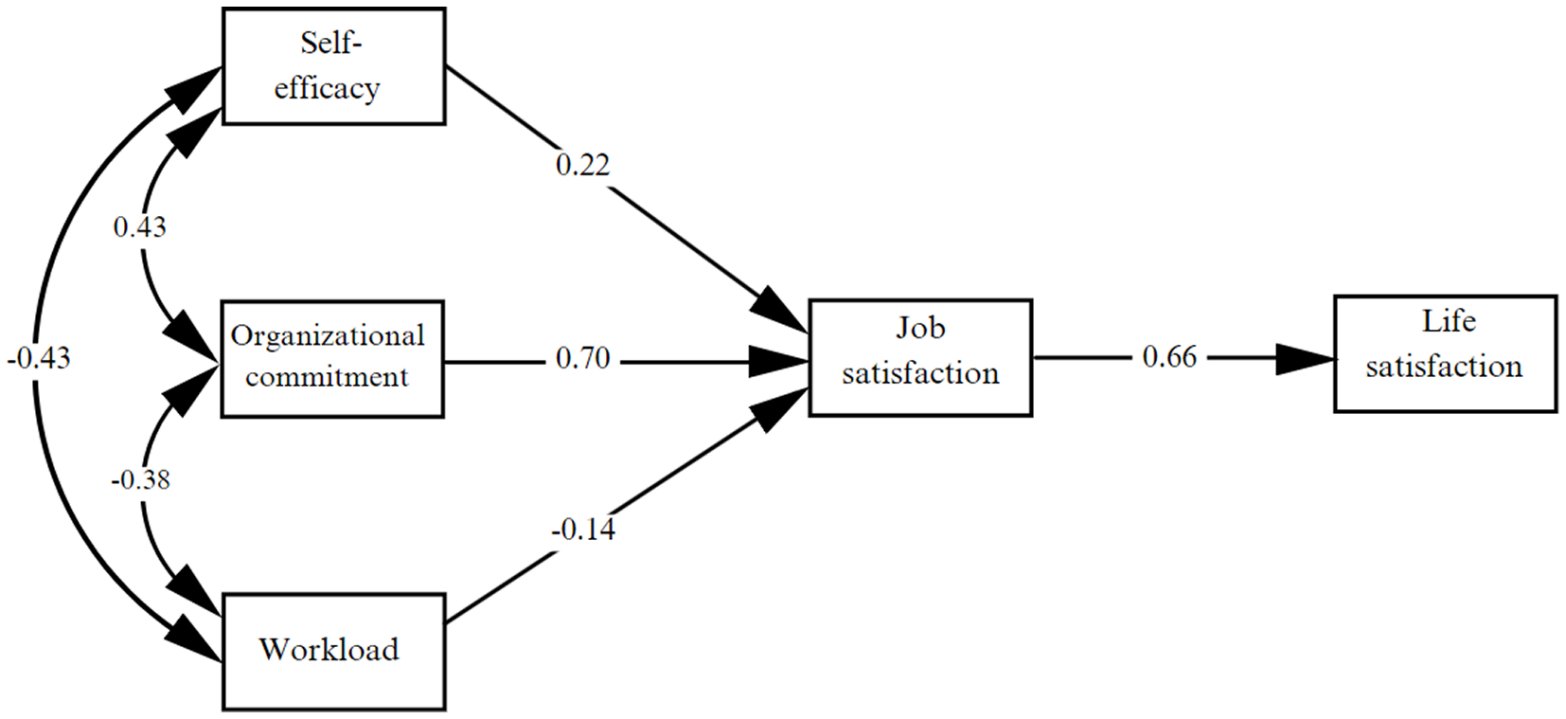
Predictive model of life satisfaction, considering the variables self-efficacy, organizational engagement and workload, and job satisfaction as mediator.

A predictive model was evaluated using structural equation modeling (Figure 2). The theoretical model analysis yielded a good fit: *χ*^2^ = 1236.28, df = 622, *p* < 0.001, CFI = 0.96, TLI = 0.96, RMSEA = 0.06 [CI: 0.05–0.06], and SRMR = 0.06. These results confirm hypotheses H1 and H2, which suggest that self-efficacy (*β* = 0.22, *p* < 0.001) and organizational commitment (*β* = 0.70, *p* < 0.001) are positive predictors of job satisfaction. Additionally, hypothesis H3, which posits that workload is a negative predictor of job satisfaction (*β* = −0.14, *p* < 0.001), is confirmed. Finally, hypothesis H4, which suggests that job satisfaction has a positive effect on life satisfaction (*β* = 0.66, *p* < 0.001), is also confirmed.

### 3.4. Mediation model

After demonstrating the good fit of the model, the resulting relationships were analyzed to verify the validity of the hypothesis and determine its predictive capacity. The model explains 49% of the total variance of life satisfaction (R2 = 49). This shows that the joint explanatory capacity is high and it is evident that the model estimates indicate a good fit since they are within the recommended value (Pérez and Medrano, 2010).

Bootstrapping with 5,000 iterations was used for the mediation analysis, and the results are shown in Table 3. The mediating role of job satisfaction in the relationship between self-efficacy and life satisfaction was confirmed, *β* = 0.13, *p* = 0.02 (H5a). The mediating role of job satisfaction between organizational commitment and life satisfaction was also confirmed, *β* = 0.57, *p* < 0.01 (H5b). Similarly, the mediation between workload and life satisfaction was confirmed, *β* = −0.11, *p* 0.01 (H5c) (Table 3).

**Table 3:** Research hypotheses on indirect effects and their estimates.

Hypothesis	Path in the model	β	*p*	95%CI	
				LL	UL
Hypothesis5a	Self-efficacy → Job satisfaction → Life satisfaction	0.13	0.02	0.04	0.27
Hypothesis5a	Organizational commitment → Job satisfaction → Life satisfaction	0.57	<0.01	0.34	0.89
Hypothesis5c	Workload → Job satisfaction → Life satisfaction	−0.11	0.01	−0.20	−0.03

## 4. Discussion

The study broadened knowledge about the research on the integrative model of job satisfaction, a model based on Duffy and Lent was tested (Lent and Brown, 2006, 2008), which describes the relationship between organizational effectiveness, work engagement, workload, job satisfaction and life satisfaction among elementary school teachers in Peru. The model had a good fit with the data and was consistent with the Duffy and Lent model in which three of the predictors (self-efficacy, organizational commitment, and workload) indicated a direct influence with job satisfaction and an indirect influence with life satisfaction, considering life satisfaction as a mediating variable. This suggests that teachers’ job satisfaction is related to their personality and to factors that contribute to the organization, in particular the ability of the teacher to contribute to the organization.

The present study has found that teachers’ self-efficacy has a positive effect on their job satisfaction. This finding is supported by previous studies that suggest that an increase in teachers’ self-efficacy is associated with greater job satisfaction (Türkoglu et al., 2017; Burić and Moè, 2020). This relationship is due in part to the fact that teachers with low self-efficacy may experience higher levels of stress, have difficulties in teaching, and present lower levels of job satisfaction (Klassen and Chiu, 2010). On the other hand, teachers with higher self-efficacy are able to improve their performance in the classroom, which translates into a better perception of teaching by their students, which in turn increases their motivation and enthusiasm (Moè et al., 2010). Self-efficacy also influences teachers’ behavior, including commitment, enthusiasm, practices, and behaviors. In this sense, to maximize the results of self-efficacy on teachers’ job satisfaction, it is important to consider factors such as support from the institution’s director, support from staff members, consensus goals, greater opportunities for personal development, induction programs, and workload reduction. This way, the teacher turnover rate can be reduced, and the retention and strength of the school environment can be improved (Viel-Ruma et al., 2010; Aldridge and Fraser, 2015).

A significant positive influence was also found between organizational commitment and teachers’ job satisfaction. This aligns with previous research suggesting that organizational commitment can influence teachers’ job satisfaction (Skaalvik and Skaalvik, 2009; Zeinabadia, 2010; Badri et al., 2013). Thus, teachers with higher organizational commitment have more willingness and efficacy in performing their duties, which contributes to their job satisfaction (Nagar, 2012; Polat and İskender, 2018). Additionally, organizational commitment can influence staff retention, as committed employees are more likely to stay in the organization and work passionately to achieve established goals (Kotzé and Nel, 2020). Organizational commitment has also been linked to academic performance and teachers’ self-efficacy, which contributes to the quality of academic performance (Park, 2007; Dee et al., 2016). In this sense, organizational commitment is an important factor in teachers’ job satisfaction. Given that organizational commitment allows teachers to have more willingness to work, greater effectiveness in performing their duties, and greater retention in the organization (Anari, 2012; Batugal and Tindowen, 2019), it is important to formulate policies that promote a high level of organizational commitment through a positive relationship, training, and addressing communication issues. Attention should also be paid to the level of commitment in younger and older teachers, and measures taken to improve their commitment to the institution and reduce teacher turnover and rotation (Yucel and Bektas, 2012).

Likewise, it has been found that workload has a negative effect on teachers’ job satisfaction, which is supported by various previous studies (Smith and Bourke, 1992; Huyghebaert et al., 2018). This effect can be explained by the fact that workload is a demand that hinders job satisfaction by generating stress and reducing teachers’ work capacity (Bakker and Bal, 2010). In addition, it has been observed that perceived workload is also relevant to teachers’ well-being and is related to emotional exhaustion and burnout (Alarcon, 2011). Furthermore, some studies have found mixed results regarding the relationship between workload and job satisfaction among teachers (Hakanen et al., 2006; Skaalvik and Skaalvik, 2007). Therefore, it is important to highlight that the type of workload may influence its effect on teachers’ job satisfaction. For example, in some studies, high levels of physical workload have been found to be negatively related to job satisfaction (Skaalvik and Skaalvik, 2007), while other studies have found that moderate mental workload benefits the maintenance and stabilization of teachers’ work capacity (Xiao et al., 2015). Therefore, it is important to take measures to reduce teachers’ workload and adjust job demands to their capabilities and needs.

On the other hand, a positive effect of job satisfaction on teachers’ life satisfaction was found. This result has been consistent with previous literature (Lent et al., 1994; Marcionetti and Castelli, 2022) indicating that job satisfaction allows teachers to be happy with their lives, progress in their work goals, and experience greater positive affect. In addition, it has been demonstrated that life satisfaction is related to a range of positive personal, psychological, and social outcomes, such as higher income, work success, better relationships, and a greater likelihood of marriage and childbirth (Gilman and Huebner, 2000; Diener and Tay, 2012). Job satisfaction is an important factor to consider when studying desirable outcomes and workers’ well-being (Locke, 1976). In the specific case of teachers, job satisfaction has been related to teacher retention and relationships with students (Veldman et al., 2013). Moreover, several studies have shown that life satisfaction is related to teachers’ job satisfaction (Lent et al., 2001). Similarly, life satisfaction depends on various factors such as health, income, spirituality, and leisure activities (Diener and Biswas-Diener, 2008). However, job satisfaction is also an important predictor of life satisfaction. This is because the characteristics of the work environment and personality influence teachers’ job satisfaction (Marcionetti and Castelli, 2022). Teachers who feel they live a meaningful life and whose values and goals are important can generate good job performance and better relationships. Teachers with higher job satisfaction are also better educational agents, as their attitudes, values, and ideas are related to their work and to an individual and collective vision (Ignat and Clipa, 2012).

Finally, it was found that job satisfaction measures the relationship between self-efficacy, job commitment, and workload in life satisfaction. These findings have important implications for practice, as they suggest that improving teacher job satisfaction can have a positive impact on their life satisfaction. In this sense, it has been found that self-efficacy directly affects goal progress, working conditions, and job satisfaction, and its effect on life satisfaction goes through job satisfaction (Lent and Brown, 2008). Likewise, it has been observed that an adequate workload can contribute to teacher job satisfaction and job performance (Bakker and Demerouti, 2017). Furthermore, job satisfaction can mediate the relationship between workload and teacher life satisfaction, which is supported by social cognitive model theories of the interaction of sources of job and life satisfaction (Marcionetti and Castelli, 2022). Previous studies also suggest that perceived workload can directly influence life satisfaction indirectly through job satisfaction (Goh et al., 2015). However, excessive workload can generate stress and reduce job satisfaction (Marcionetti and Castelli, 2022). Therefore, it is important to take measures to reduce teacher workload and adjust job demands to their capacities and needs. The results obtained confirm the importance of job satisfaction as a mediator in the relationship between job variables and life satisfaction. Further research is required in this line to better understand the mediating processes between job variables and life satisfaction of elementary school teachers. Additionally, special attention should be paid to workload and its influence on teacher job satisfaction, in order to adjust job demands to their capacities and needs, and thus improve their overall well-being.

### 4.1. Implications

Understanding the motivations of teachers to develop new tasks and practices in their careers is necessary. Therefore, organizations must provide greater opportunities for development, working conditions, promotion, suitable organizational environment, and greater autonomy to provide better quality of care in teaching-learning environments. Professional development programs allow teachers to gain more confidence, improve their skills and knowledge, which increases their self-efficacy and job satisfaction while reducing work stress. Attention should also be paid to the workload of basic education teachers, as it can directly influence their satisfaction with life indirectly through job satisfaction. Therefore, it is essential to adjust job demands to the abilities and needs of teachers to avoid excessive workload that can generate stress and reduce their job and life satisfaction. Moreover, it is important to foster the self-efficacy and organizational commitment of teachers, as these factors have a positive relationship with their life satisfaction through job satisfaction. In this regard, it is essential to provide opportunities for professional development and emotional support to teachers to improve their self-efficacy and commitment to work, thus increasing their job and life satisfaction. Finally, it is necessary to consider that job satisfaction is not sufficient to guarantee the well-being of teachers, as burnout is a negative indicator of the same. Therefore, preventive measures must be implemented to avoid teacher burnout, such as promoting a healthy work environment, fostering emotional resilience, and implementing fair and equitable labor policies.

### 4.2. Limitations

The results of this study should be considered with some limitations. First, because it was a cross-sectional study, the data cannot infer causality, and future studies should use a longitudinal or experimental design to analyze in depth the causal mechanisms underlying the interactions of the variables studied. Second, since the sample was made up of elementary school teachers, it is possible that the findings cannot be generalized to other populations or regions. Third, the self-report method performed by teachers is loaded with tendencies that link the responses to remembered experiences, thus, future studies should ensure that teachers report on the study variables after some program that will increase the accuracy of the reports. Fourth, teachers voluntarily participated in the study and it is possible that teachers with lower self-efficacy, work engagement, job satisfaction, life satisfaction, and teachers with higher workloads opted not to participate, which could lead to biased estimates and range restrictions. However, future studies could choose to improve the methodology and collection of data and the collection of data at corporate meetings to broaden the overall response rate.

## 5. Conclusion

The importance of self-efficacy, organizational commitment, and workload in job satisfaction and, in turn, life satisfaction of basic education teachers is confirmed. The findings support Lent and Brown’s (2006) sociocognitive theories and demonstrate how job satisfaction acts as an important mediator in this relationship. It is important to note that job satisfaction not only has a direct effect on life satisfaction but also has an indirect effect through self-efficacy, organizational commitment, and workload. These results will enable educational organizations that seek to improve the well-being and satisfaction of their teachers. In particular, measures should be taken to reduce the workload of teachers and adjust job demands to their abilities and needs, promote self-efficacy and organizational commitment, and foster a healthy and sustainable work environment. Additionally, professional development programs and emotional regulation may be beneficial in improving job satisfaction and reducing job stress.

## Data availability statement

The raw data supporting the conclusions of this article will be made available by the authors, without undue reservation.

## Ethics statement

The studies involving human participants were reviewed and approved by Research Ethics Committee of Universidad Peruana Unión. The patients/participants provided their written informed consent to participate in this study.

## Author contributions

JC, WM-G, and JS designed the study. DB-M, AP, FA, and KP wrote the first draft of the manuscript. DB-M, AP, JC, WM-G, and JS analyzed and interpreted the data. All authors contributed to the article and approved the submitted version.

## Conflict of interest

The authors declare that the research was conducted in the absence of any commercial or financial relationships that could be construed as a potential conflict of interest.

## Publisher’s note

All claims expressed in this article are solely those of the authors and do not necessarily represent those of their affiliated organizations, or those of the publisher, the editors and the reviewers. Any product that may be evaluated in this article, or claim that may be made by its manufacturer, is not guaranteed or endorsed by the publisher.
